# Branched Chondroitin Sulfate Oligosaccharides Derived from the Sea Cucumber *Acaudina molpadioides* Stimulate Neurite Outgrowth

**DOI:** 10.3390/md20100653

**Published:** 2022-10-21

**Authors:** Weili Wang, Hui Mao, Sujuan Li, Longlong Zhang, Lian Yang, Ronghua Yin, Jinhua Zhao

**Affiliations:** 1 State Key Laboratory of Phytochemistry and Plant Resources in West China, Kunming Institute of Botany, Chinese Academy of Sciences, Kunming 650201, China; 2 University of Chinese Academy of Sciences, Beijing 100049, China; 3 State Key Laboratory for Conservation and Utilization of Bio-Resources, Key Laboratory for Microbial Resources of the Ministry of Education, School of Life Sciences, Yunnan University, Kunming 650091, China; 4 School of Pharmaceutical Sciences, South-Central Minzu University, Wuhan 430074, China

**Keywords:** fucosylated chondroitin sulfate, sea cucumber, disaccharide branch, oligosaccharide, neurite outgrowth, stimulation

## Abstract

Fucosylated chondroitin sulfate (FCS) from the sea cucumber *Acaudina molpadioides* (FCS*_Am_*) is the first one that was reported to be branched by disaccharide GalNAc-(α1,2)-Fuc_3S4S_ (15%) and sulfated Fuc (85%). Here, four size-homogenous fractions, and seven oligosaccharides, were separated from its β-eliminative depolymerized products. Detailed NMR spectroscopic and MS analyses revealed the oligomers as hexa-, hepta-, octa-, and nonasaccharide, which further confirmed the precise structure of native FCS*_Am_*: it was composed of the CS-E-like backbone with a full content of sulfation at O-4 and O-6 of GalNAc in the disaccharide repeating unit, and the branches consisting of sulfated fucose (Fuc_4S_ and Fuc_2S4S_) and heterodisaccharide [GalNAc-(α1,2)-Fuc_3S4S_]. Pharmacologically, FCS*_Am_* and its depolymerized derivatives, including fractions and oligosaccharides, showed potent neurite outgrowth-promoting activity in a chain length-dependent manner. A comparison of analyses among oligosaccharides revealed that the sulfate pattern of the Fuc branches, instead of the heterodisaccharide, could affect the promotion intensity. Fuc_2S4S_ and the saccharide length endowed the neurite outgrowth stimulation activity most.

## 1. Introduction

**Figure 1:**
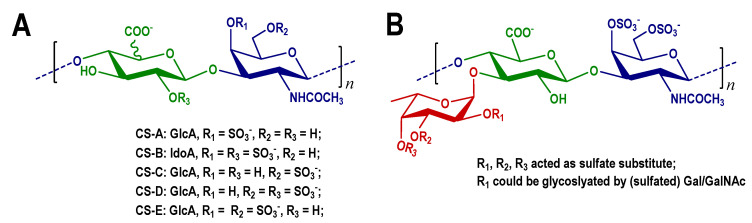
Structures of typical CS (**A**), and FCS from sea cucumbers (**B**).

Neurons are highly differentiated terminal cells that cannot regenerate. If compounds can promote neuronal growth, it will be meaningful for treatment of neuronal damage and degeneration. Chondroitin sulfate (CS) is a class of glycosaminoglycans and is involved in cell division and neuronal development. CS proteoglycans (CS-PGs) were defined as inhibitory molecules of neuron growth in early experiments, and the use of chondroitinase ABC could attenuate the inhibitory effect on neuron growth [1,2,3]. However, some researches have reported that CS could promote neuronal growth [4]. According to the sulfation sites on the saccharide chain, CS could be classified as type A, B, C, D, E, et al. (Figure 1A). CS-A, CS-B, and CS-C were considered to inhibit neuron growth, while CS-D and CS-E exhibited neurite outgrowth-promoting activity. Some reports also proved that CS-E strongly inhibited nerve regeneration [5,6,7,8,9,10]. It has been known that commercially available CS retains heterogeneity, including CS-A (50–80% of A-type unit), CS-C (50–70% of C-type unit), CS-D (20–40% of D-type unit) and CS-E (63.6% of E-type unit). The low purity of saccharides may contribute to the contradictory results [11].

In regard to the CS analogs from invertebrates, the keratan sulfate disaccharide-branched CS-E from a clam, *Mactra chinensis*, stimulated the neurite outgrowth of hippocampal neurons [11]. A fucosylated CS from the sea cucumber *Apostichopus japonicus* exhibited neurite outgrowth-promoting activity [12].

Fucosylated CS (FCS) is a characterized CS-E derivative that has been found ubiquitously and exclusively in sea cucumbers thus far. It possesses the CS-E-like backbone with repeating disaccharide units [D-GalNAc_4S6S_-β(1,4)-D-GlcA-β(1,3)], where D-GalNAc are sulfated in O-4 and O-6 sites [13]. The O-3 of D-GlcA in each backbone are branched by sulfated fucose or disaccharide [14,15,16,17,18,19]. Structural differences of FCS from different sea cucumber species are embodied in the size, the composition, the sulfate substitution of branches (Figure 1B). Besides structure, another aspect of FCS that appeals to researchers is that FCS has wide-ranging biological activities, such as anti-coagulant, anti-thrombotic, anti-virus, and anti-cancer effects [13,20,21,22]. Previous research proposed that the FCS from the sea cucumber *A. japonicus* exhibited neurite outgrowth-promoting activity, and recognized the potential roles of the fucose branch in neurite outgrowth. A chemically synthesized FCS trisaccharide that is composed of a CS-E disaccharide unit with a sulfated fucose branch also exhibited comparable stimulation activity [12]. The effects of the FCS originated from other sea cucumbers on neurite’s growth, and the structure-function relationships remain unresolved and deserve detailed investigations.

FCS*_Am_* was obtained from the sea cucumber *Acaudina molpadioides* in our previous work [23]. Oligosaccharides from the β-eliminative depolymerized FCS*_Am_* revealed that its backbone has a full content of type E, which was branched by monosaccharide [Fuc_2S4S_ and Fuc_4S_], and the disaccharide [GalNAc-(α1,2)-Fuc_3S4S_] on O-3 of each GlcA. This is the first report of such a disaccharide branch in natural FCS. FCS*_Am_* showed the variety of branches with sulfated substitutions, as well as the novel disaccharide.

In this work, further investigation on this unique CS analog (FCS*_Am_*) was conducted. From its β-eliminative depolymerized products, the size-homogenous fractions with a higher degree of polymerization were obtained. Subsequently, charge-separation by SAX-HPLC was employed to yield hexa-, hepta-, octa-, and nonasaccharide. These oligosaccharides clearly revealed the structural features of natural FCS*_Am_*, and further verified the characteristics of its branches which is in line with our previous report. In view of the novelty of FCS*_Am_*, and the structural diversity of its derivatives, including the size-homogenous fractions and oligosaccharides, the effects on neurite outgrowth were evaluated and the structure-activity relationships were also discussed.

## 2. Results and Discussion

### 2.1. Preparation of Homogenous Fractions and Oligosaccharides from the Depolymerized FCS_Am_

**Figure 2:**
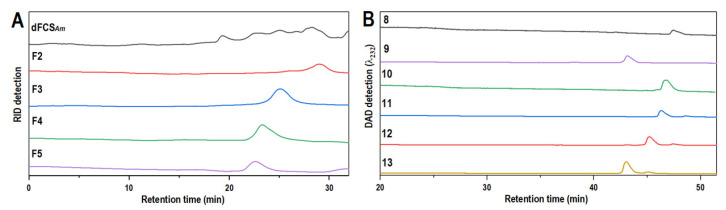
HPGPC profiles of dFCS*_Am_*, F2–F5 (Superdex peptide 10/300 GL column) (**A**); HPLC profiles of oligosaccharides **8**–**13** (Dionex IonPac™ AS11-HC column) (**B**).

β-Eliminative depolymerization has been established as an effective method for deciphering the structures of FCS from sea cucumbers. Here the FCS*_Am_* was employed as the depolymerization process described in Section 3.2 to prepare its low-molecular-weight product, dFCS*_Am_*. As shown in Figure 2A, dFCS*_Am_* was composed of a series of fractions with different sizes, reflected by its HPGPC profile. For the sake of their structural identification, dFCS*_Am_* was fractionated by GPC using Bio-Gel P-10 & P-6 columns, until each fraction exhibited a relatively single and symmetric peak on the HPGPC analysis chromatogram. Finally, five fractions (F1–F5) were obtained (Figure 2A).

F1 and F2 have been further analyzed in our previous work. Both fractions consisted of the oligomers with the same or approximate size. From F1, three trisaccharides were obtained. And from F2, two tetra-, one penta-, and two hexa-saccharides were purified by the aid of SAX separation (oligosaccharides **1**–**7**). For the fractions with larger size, theoretically, its oligosaccharide composition should be more complex and variable. Though F3–F5 presented the homogeneities in size, further analysis based on the charge difference showed the characteristic of a multi-component. In this work, F3 was further charge-separated on the Dionex Ionpac™ AS11-HC Semi-prep column repeatedly to yield oligosaccharides **8**–**13**. Chromatographic analysis indicated that **8**–**13** running over the SAX column presented a single chromatographic peak, respectively (Figure 2B). The same separation strategy has been adopted for fraction F4, while, unfortunately, the separated components are mixtures as well.

### 2.2. Structural Elucidation of the Oligosaccharides

**Figure 3:**
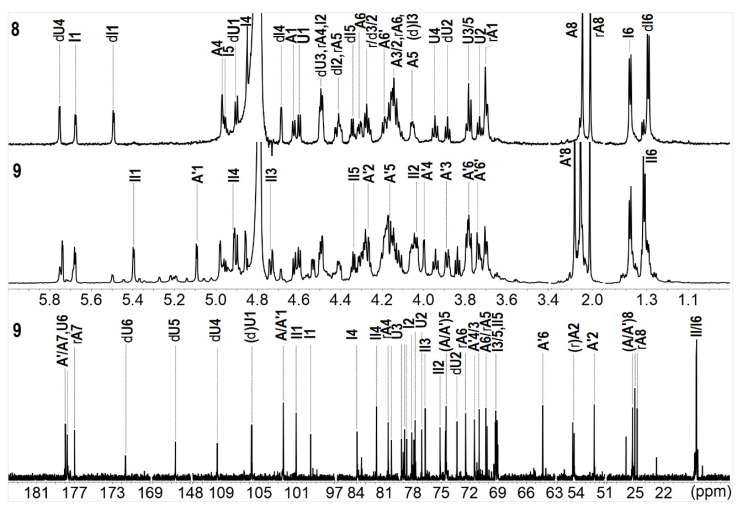
1D NMR spectra and chemical shifts assignments of **8** and **9**.

**Figure 4:**
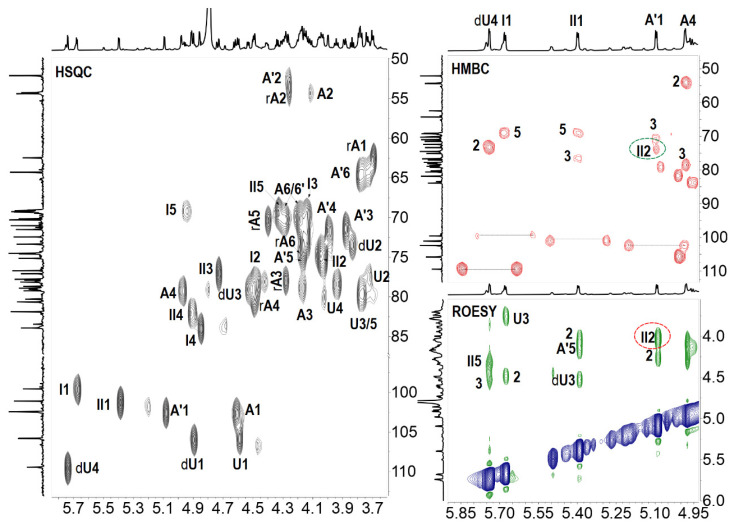
^1^H-^13^C HSQC, partial ^1^H-^13^C HMBC, ^1^H-^1^H ROESY spectra and signal assignments of **9**.

**Figure 5:**
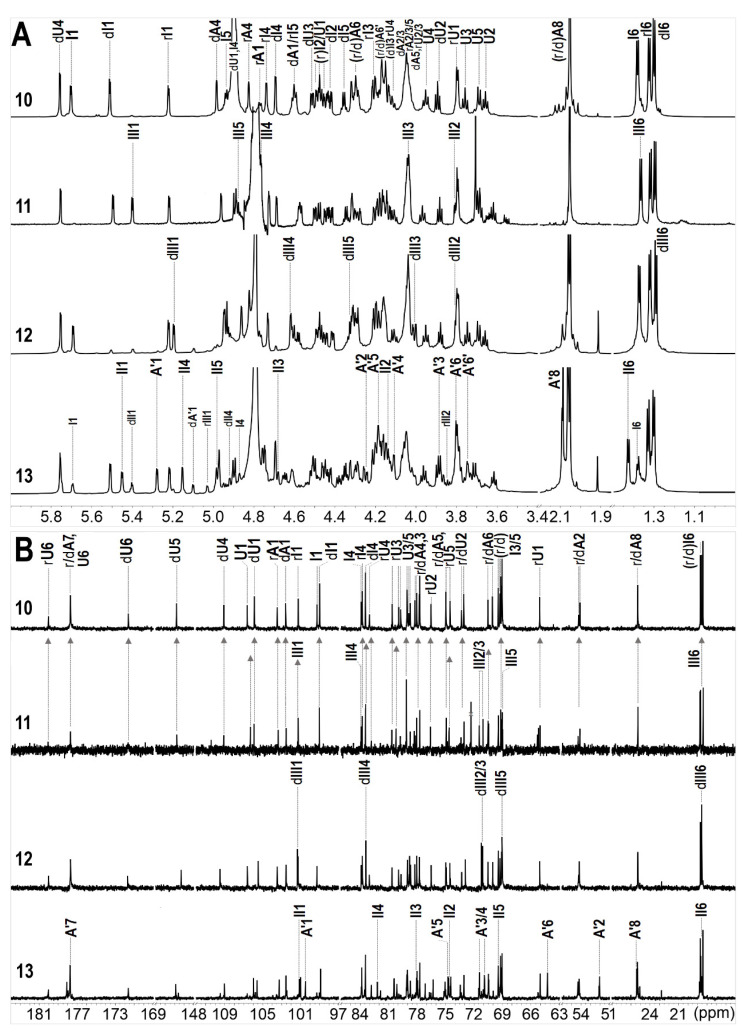
1D (^1^H, (**A**); ^13^C, (**B**)) NMR spectra and chemical shifts assignments of **10–13**.

**Figure 6:**
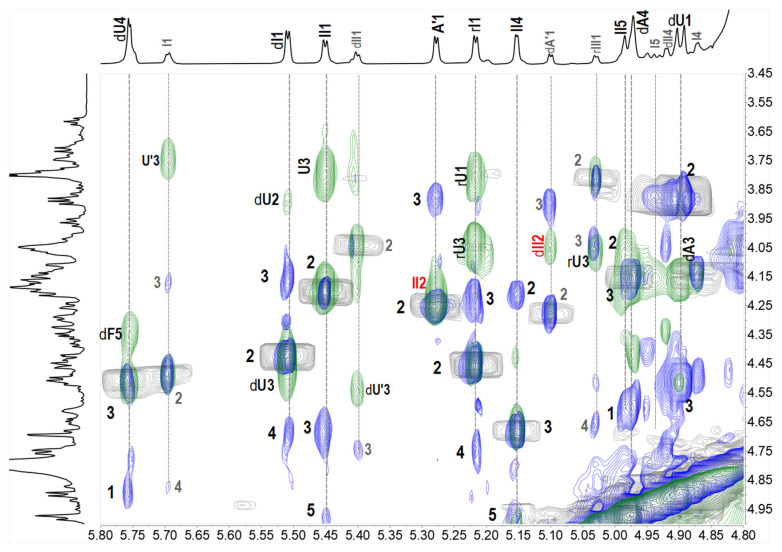
Partial superimposed spectra of ^1^H-^1^H COSY/TOCSY/ROESY of **13** (COSY-gray, TOCSY-blue, ROESY-green).

The structures of oligosaccharides **8**–**13** were elucidated by the aid of their 1D & 2D NMR spectra (Figure 3, Figure 4, Figure 5 and Figure 6, and Figures S2 and S3) and ESI-Q-TOF MS spectra (Figure S5).

The spectra of compound **8** indicated it was a hexasaccharide with the structure of L-Fuc_2S4S_-(α1,3)-L-Δ^4,5^GlcA-(α1,3)-D-GalNAc_4S6S_-(β1,4)-[L-Fuc_2S4S_-(α1,3)-]-D-GlcA-(β1,3)-D-GalNAc_4S6S_-ol, the same as the one we have reported before [18]. The distinctive methyl groups signaling at ~2.0 ppm (-COCH_3_) and ~1.3 ppm (-CH_3_) in the ^1^H NMR spectrum revealed that **8** contained two GalNAc residues and two Fuc residues. The anomeric protons at *δ*_H_ 5.68 and 5.50 ppm, derived from the two Fuc residues, indicated the sulfation at O-2 and O-4 positions. The downfield shift signals of the respective protons (I2, 4.49 ppm; I4, 4.85 ppm; dI2, 4.42 ppm; dI4, 4.69 ppm) reconfirmed the substitution of sulfate esters. For oligosaccharide **9**, as shown in Figure 3, the appearance of one more set of anomeric signals (at the region of *δ*_H_ 5.0–5.7 ppm, *δ*_C_ 100–106 ppm), accompanying with one more methyl signal at *δ*_H_ 2.09 ppm, indicated that **9** possessed one more GalNAc residue (marked as A’) than that in **8**. Another signal at *δ*_H_ 5.40 ppm was from the anomeric proton of Fuc residue (designated as II) with the sulfation at O-3 and O-4 positions, which were confirmed by the resonance signals (II3: 4.74, 76.7 ppm; II4: 4.91, 81.9 ppm). Every resonance of each residue was determined by the HSQC spectrum (Figure 4). The correlation of A’1 (*δ*_H_ 5.09 ppm) and II2 (*δ*_C_ 75.1 ppm) in the HMBC spectrum indicated that residue A’1 connected to II by an α1,2 linkage, and this linkage could be reconfirmed by the correlation of A’1 (*δ*_H_ 5.09 ppm) and II2 (*δ*_H_ 4.04 ppm) in the ROESY spectrum (Figure 4). Thus, **9** was proved to be a heptasaccharide with the structure of D-GalNAc-(α1,2)-L-Fuc_3S4S_-(α1,3)-L-Δ^4,5^GlcA-(α1,3)-D-GalNAc_4S6S_-(β1,4)-[L-Fuc_2S4S_-(α1,3)-]-D-GlcA-(β1,3)-D-GalNAc_4S6S_-ol.

The 1D spectra of **10**–**12** occurred similarly in the number of characteristic signals, including the methyl groups (two -COCH_3_ of GalNAc, *δ*_H_~2.0 ppm, and three -CH_3_ of Fuc, *δ*_H_~1.3 ppm), the anomeric resonances (three signals, at *δ*_H_ 5.0–5.7 ppm; seven signals at *δ*_C_ 99–107 ppm), and the two carbon signals of C-2 of GalNAc residues (*δ*_C_~54 ppm) (Figure 5). This suggested that **10**–**12** shared the common skeleton of an octasaccharide. Detailed analyses based on their 2D NMR spectra lead to the full assignments of all the signals, which are shown in Figure 5 and Table 1. The structural differences between **10**–**12** existed in the sulfation form of the Fuc branches. According to the downfield shift signals of H2/C2 and H4/C4 (marked in Italic, Table 1), three Fuc_2S4S_ (dI, I, and rI represented the Fuc_2S4S_ locating at the non-reducing end, the middle, and the reducing end, respectively) were determined in oligosaccharide **10**, while in **11** and **12**, an Fuc_4S_ at the middle and the non-reducing end were elucidated and designated as III and dIII, respectively. The structures of **10**–**12** were determined as L-Fuc_2S4S_-(α1,3)-L-Δ^4,5^GlcA-(α1,3)-D-GalNAc_4S6S_-(β1,4)-[L-Fuc_2S4S_-(α1,3)-]-D-GlcA-(β1,3)-D-GalNAc_4S6S_-(β1,4)-[L-Fuc_2S4S_-(α1,3)-]-D-GlcA-ol, L-Fuc_2S4S_-(α1,3)-L-Δ^4,5^GlcA-(α1,3)-D-GalNAc_4S6S_-(β1,4)-[L-Fuc_4S_-(α1,3)-]-D-GlcA- (β1,3)-D-GalNAc_4S6S_-(β1,4)-[L-Fuc_2S4S_-(α1,3)-]-D-GlcA-ol, and L-Fuc_4S_-(α1,3)-L-Δ^4,5^GlcA-(α1,3)-D-GalNAc_4S6S_-(β1,4)-[L-Fuc_2S4S_-(α1,3)-]-D-GlcA-(β1,3)-D-GalNAc_4S6S_-(β1,4)-[L-Fuc_2S4S_-(α1,3)-]-D-GlcA-ol. The full assignments are displayed in Table 1. The structures of **8**–**12** were also confirmed according to the accurate molecular mass information of the excimer ion peaks, as shown in Table 2.

**Figure 7:**
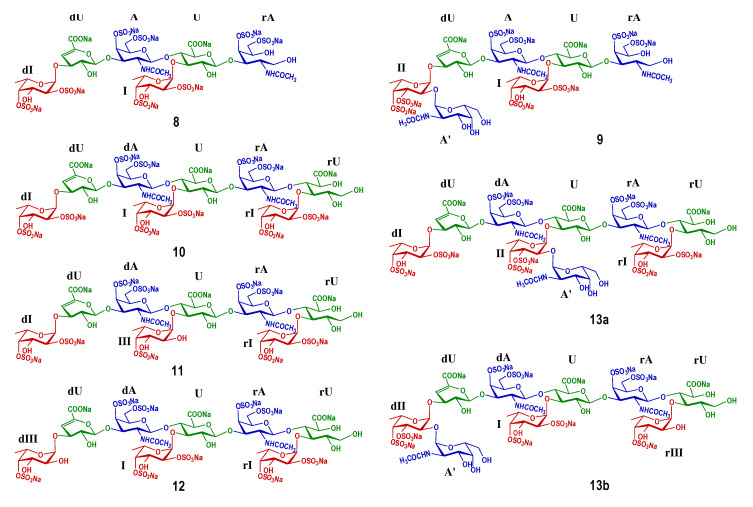
Structures of oligosaccharides **8**–**13**.

For **13**, two sets of signals different in the integration area reflected in its ^1^H NMR spectrum, suggesting that **13** was a mixture. In terms of the prevailing component, compared with **10**, three more signals were observed at the region of 5.0–5.7 ppm besides the H-1 of Fuc_2S4S_ at the non-reducing and reducing ends (5.51 and 5.22 ppm). According to the superimposed spectra of ^1^H-^1^H COSY/TOCSY/ROESY of **13** (Figure 6), the protons at 5.15 and 5.45 ppm came from the same spin coupling system. Detailed analysis on the COSY and TOCSY spectra showed that it was an Fuc residue with sulfation at O-3 and O-4 (Fuc_3S4S_, designated as II). The H-5 of II (4.98 ppm) indicated that it is in the middle of the saccharide chain. The proton at 5.28 ppm, combining with the extra carbon signal at 100.5 ppm, and the extra methyl signal at ~2.0 ppm, outlined the presence of a GalNAc residue (A’). A’ connected to II by an α1,2 linkage, revealing from the correlation peak of A’1 (5.28 ppm) and II2 (4.15 ppm) in the ROESY spectrum. Thus, the main component in **13** (designated as **13a**) was deduced as a nonasaccharide with the structure of L-Fuc_2S4S_-(α1,3)-L-Δ^4,5^GlcA-(α1,3)-D-GalNAc_4S6S_-(β1,4)-[D-GalNAc-(α1,2)-L-Fuc_3S4S_-(α1,3)-]-D-GlcA-(β1,3)-D-GalNAc_4S6S_-(β1,4)-[L-Fuc_2S4S_-(α1,3)-]-D-GlcA-ol. For the minor component, it was also elucidated as a nonasaccharide with the structure of D-GalNAc-(α1,2)-L-Fuc_3S4S_-(α1,3)-L-Δ^4,5^GlcA-(α1,3)-D-GalNAc_4S6S_-(β1,4)-[L-Fuc_2S4S_-(α1,3)-]-D-GlcA-(β1,3)-D-GalNAc_4S6S_-(β1,4)-[L-Fuc_4S_-(α1,3)-]-D-GlcA-ol (**13b**). The structures of oligosaccharides **10**–**13** were presented in Figure 7.

### 2.3. Structural Confirmation of the Native FCS_Am_

**Figure 8:**
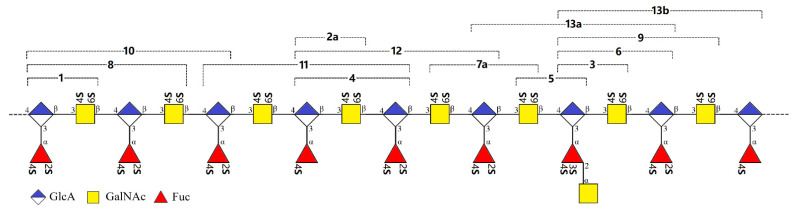
Diagram of the structure of FCS*_Am_* and the oligosaccharides obtained through β-elimination depolymerization. The structures of **1**–**7** were presented in the literature [23].

In our last work, from the size-homogeneous fractions with low degree of polymerization that separated from the β-eliminative depolymerized product dFCS*_Am_*, we have elucidated a series of purified oligosaccharides, including tri-, tetra-, penta-, and hexasaccharides [23]. The branches contained the mono- and disaccharide [Fuc_4S_, Fuc_2S4S_, and GalNAc-(α1,2)-Fuc_3S4S_]. Here, we have purified and elucidated the oligomers **8**–**13** consisting of hexa-, hepta-, octa-, and nonasaccharide from the fraction **F3** with a higher degree of polymerization. **8**–**13** presented a continuation with the structural features summarized before which are composed of the central core of 3)-D-GalNAc_4S6S_-(β1,4)-D-GlcA-(β1, and the sulfated branches [Fuc_4S_, Fuc_2S4S_, and GalNAc-(α1,2)-Fuc_3S4S_]. Oligosaccharides that contain the heterodisaccharide branch were confirmed again. These results further supported our structural deduction of the natural FCS*_Am_*: its backbone consists of a repeating disaccharide unit of GlcA and GalNAc linked alternatively by β1,4 and β1,3, wherein all the GalNAc residues were sulfated at O-4 and O-6, the same as CS-E; all the GlcA residues were branched and all the branches glycosylated at O-3 of GlcA, instead of any other sites; the branch types included sulfated fucose (Fuc_4S_ and Fuc_2S4S_) and heterodisaccharide [GalNAc-(α1,2)-Fuc_3S4S_]; the disaccharide branches distributed randomly, but there was no case that such disaccharide branches adjacently distributed in the saccharide chain. All of the reducing end residues (Fuc) of the heterodisaccharide branch were sulfated both at O-3 and O-4, while no sulfation occurred on any sites of the non-reducing end residues (GalNAc) (Figure 8). The sulfation pattern of the heterodisaccharide branch seemed conserved, markedly differing from that reported in the FCS from the sea cucumbers *Holothuria nobilis* and *Ludwigothurea grisea* [17,19].

### 2.4. Neurite Outgrowth-Promoting Activity of FCS_Am_ and Its Derivatives

FCS*_Am_* and a variety of its derivatives, including the size-homogenous fractions with different molecular weights and the purified oligosaccharides with different sulfate substitutions, laid the substance that is fundamental for the further analysis of the influence of structural characteristics on the neurite outgrowth-promoting activity.

**Figure 9:**
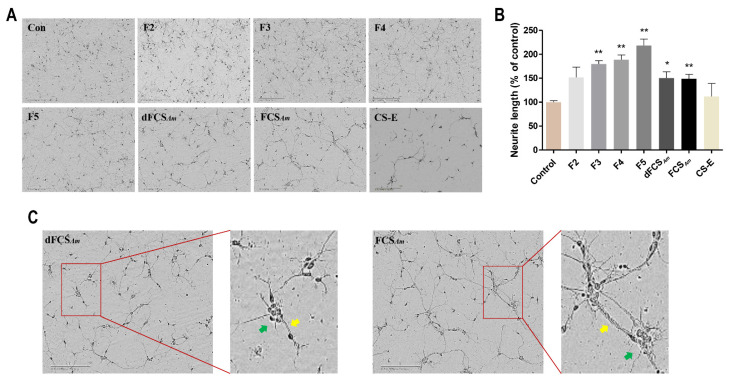
Effects of FCS*_Am_*, dFCS*_Am_*, and the size-homogeneous fractions F2–F5 on neurite outgrowth (*n* = 4–6) (**A**,**B**). Cells were cultured for 2 days in plates coated with PDL (50 μg/mL) alone (control) or size-homogeneous fractions F2–F5 (50 μg/mL). The length (*n* = 50–100 cells) of the longest neurite (mean ± SD) was measured by Image J software and expressed as percentage of growth relative to control (* *p* < 0.05, ** *p* < 0.01); the aggregation phenomenon of FCS*_Am_*, and dFCS*_Am_*, and neurite and cell body aggregation are indicated by yellow and green arrows, respectively (**C**). Scale bar, 200 μm.

As shown in Figure 9 and Table 3, natural FCS*_Am_* and its depolymerized product dFCS*_Am_* showed significant activity in promoting neurite outgrowth. Compared with blank control and CS-E groups, the length increased by 50% at the concentration of 50 μg/mL. Meanwhile, FCS*_Am_*, dFCS*_Am_* and CS-E showed an aggregation phenomenon under the condition (Figure 9C). While for the size-homogenous fractions F2–F5 obtained after GPC treatment, they all showed the promotion activities, and the promoting effects increased with the increase of molecular weight from 1.5 to 4.4 kDa. Among them, F5 exhibited more than two-times the outgrowth promoting activity than CS-E, and no obvious aggregation phenomenon occurred. In contrast, FCS*_Am_* did not show a significantly stronger growth promoting effect than fractions, as expected, indicating that an optimum chain length is required for promotion. For dFCS*_Am_*, the presence of the saccharides with large Mw (Figure 2A) should be responsible for the weaker promotion effect than F5.

**Figure 10:**
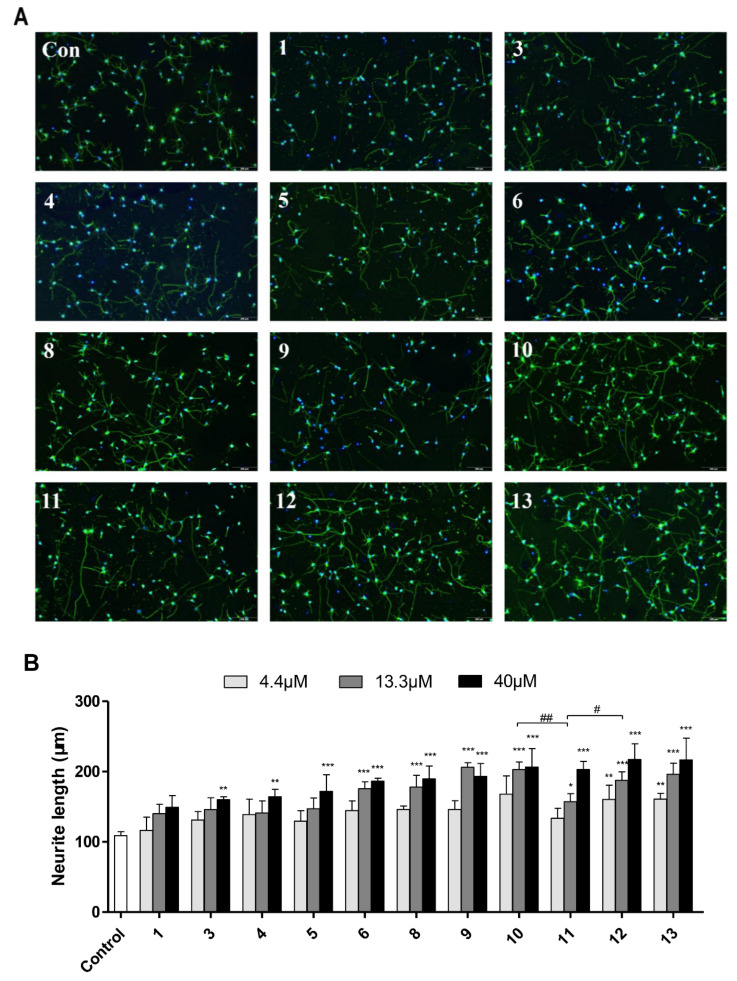
Effects of oligosaccharides **1**, **3**–**6**, and **8**–**13** from dFCS*_Am_* on neurite outgrowth (*n* = 3). Neurons were cultured in 96 plates for 2 days in plates coated with PDL (50 μg/mL) alone (control) or with oligosaccharides. The neurons were fixed and visualized with 4% paraformaldehyde and TritonX-100, and incubated with tublin _Ш_ (green) and DAPI (blue) (**A**). The length (*n* = 50–100 cells) of the longest neurite (mean ± SD) was measured by Image J software and expressed as length, relative to control (*, ^#^ *p* < 0.05, **, ^##^ *p* < 0.01, or *** *p* < 0.001) (**B**). Scale bar, 200 μm.

For the purified oligosaccharides from tri- to nonasaccharides, different concentrations (4.4, 13.3, 40 μM) of oligosaccharides were coated on PDL to evaluate the effects. As shown in Figure 10 and Table 4, compared with the control, all of the oligosaccharides showed neurite outgrowth-promoting activity to different degrees, which basically acted in a concentration-dependent manner. In response to the trisaccharide **1**, the length of neurons increased by approximately 50%, which was consistent with the previous report [12]. In addition, at the same concentration, the activity of oligosaccharides on neuron growth increased with the increase of the chain length. By comparing the structural characteristics and activity effects of **10**, **11** and **12**, the sulfate ester enhanced the activity at low (4.4 μM) and medium (13.3 μM) concentrations. Moreover, the residue Fuc_2S4S_, instead of Fuc_4S_, located at the middle of octasaccharide (**10**–**12**) showed greater promotion. By contrast, whether the branch located at non-reducing end is Fuc_2S4S_ or Fuc_4S_, the octasaccharides (**10** and **12**) showed a similar enhancement effect on neurite outgrowth. For the novel disaccharide branch [GalNAc-(α1,2)-Fuc_3S4S_], it did not show an obvious difference in the promotion or inhibition effects, which could be concluded from the comparison of analyses of **1** & **3**, **8** & **9** and **10**–**13**.

## 3. Materials and Methods

### 3.1. Materials

Fucosylated chondroitin sulfate from the body walls of *A. molpadioides* were obtained in our previous work. Bio-Gel P-10/P-6/P-2 and Sephadex G-25 were purchased from Bio-Rad Laboratories and GE Healthcare Life Sciences, respectively. CS-E [60% of E-type unit, Code# CSR-NaCS-E2(SqC)10] was bought from Cosmo Bio Co., Ltd. Neurobasal (21103049), B27 (17504044), glutamax (35050061), penicillin and streptomycin (15140122) were obtained from Gibco. Poly-D-lysine (PDL, P6407), DNase I (D4513) Anti-tublin _Ш_ (T8578) and Bovine serum albumin (BSA, B2064) were purchased from Sigma. PBS (092810305) and Triton X-100 (QR12914) are from MP. Papain (LS003119) was obtained from Worthington. Alexa flour 488 anti-mouse IgG (ab15105) came from Abcam. Pregnant Sprague-Dawley rats were obtained from SPF (Beijing) Biotechnology Co. Ltd. (Beijing, China). All other reagents were of analytical grade and obtained commercially.

### 3.2. Depolymerization of FCS_Am_

The β-eliminative depolymerization of FCS*_Am_* was employed according to an established method. Specifically, 6.5 g FCS*_Am_* was dissolved in 97.5 mL of water and then reacted with 245 mL of benzethonium chloride solution (62.5 mg/mL) under stirring at room temperature. After centrifugation (4000 rpm × 15 min) and dryness under reduced pressure at 40 °C to constant weight, 17.5 g benzethonium salts of FCS*_Am_* were obtained. They were then dissolved in 88 mL dimethyl formamide, and 6.23 mL benzyl chloride was added at 35 °C for 24 h. After that, 53.7 mL of freshly prepared EtONa/EtOH (0.16 M) was added into the reaction system for another 30 min at room temperature at 300 rpm. Subsequently, 120 mL of saturated sodium chloride solution and 960 mL EtOH were added and the precipitate was collected by centrifugation. In order to remove the benzyl groups, the resulting precipitate was dissolved in 360 mL H_2_O, and 16 mL freshly prepared 6M NaOH was added into the solution at 25 °C for 30 min. Meanwhile, 1385 mg of sodium borohydride was added to the solution to reduce the hemiacetals of the resulting saccharides. After that, the solution was neutralized by HCl. Finally, the β-eliminative depolymerized product was obtained after desalting by Sephadex G-25.

### 3.3. Preparation of Size-Homogenous Fractions and Oligosaccharides from Depolymerizated FCS_Am_

dFCS*_Am_* was subjected to a Bio-Gel P-10 column and eluted by 0.2 M sodium chloride solution in batches. The sample in tubes was detected at the UV wavelength of 232 nm, and according to the absorption values, the elution curve was plotted. After repeating separation using Bio-Gel P-10 or P-6 column, the fractions with a similar size were pooled and lyophilized separately. The obtained five size-homogenous fractions were designated as F1~F5 in order of size from lowest to highest.

F3 was further purified by strong anion exchange chromatography using a Dionex Ionpac AS11-HC Semi-prep column. The gradient elution program was as follows: 0−60 min, 0−60% elution B (2 M NaCl, pH 3.5); elution A was H_2_O (pH 3.5). The flow rate was 3.5 mL/min. The preparation was employed under the online UV detection at λ_232 nm_. Next, the oligosaccharides were desalted by GPC on a Bio-Gel P-2 or Sephadex G-25 column and lyophilized.

### 3.4. Structural Elucidation of the Oligosaccharides

The structures of the oligosaccharides were determined by 1D & 2D NMR spectra and ESI-Q-TOF-MS data, which were recorded at 298 K with a Bruker Advance 600/800 MHz spectrometer equipped with a ^13^C/^1^H dual probe in FT mode, and with a 6540 UHD Accurate-Mass Q-TOF LC/MS spectrometer. For the NMR data acquisition, each sample was dissolved in deuterium oxide (D, 99.9%).

### 3.5. Neuronal Cultures and Coating Plates

Neurobasal medium contained B27 supplement (1×), 2 mM glutamax, penicillin and streptomycin (1×). The plates were pre-coated with 50 μg/mL PDL at 37 °C for 6 h, washed three times with double distilled water and then coated with the sample overnight at 37 °C. The cortical tissue of rats at embryonic day (E) 18 were isolated under a dissecting microscope, removed quickly, and placed in cold PBS and gently chopped, then incubated in a 1 mg/mL papain and DNase I (20 IU/mL) solution at 37 °C for 30 min. Supplementing with neurobasal medium, the cells were eventually plated at 10,000/cm^2^ into plates. The cultures were maintained at 37 °C in a humidified 5% CO_2_ atmosphere [24,25,26].

### 3.6. Immunofluorescence

After 2 days, neurons were fixed with 4% paraformaldehyde for 30 min, washed with PBS three times, incubated in 0.2% Triton X-100 for 15 min, blocked with 1% BSA and incubated overnight with anti-tublin _Ш_ antibody at 4 °C. The second day, the cells were washed in PBS three times. The second antibody was then incubated at room temperature for 1 h and then rinsed in PBS three times. The nuclei were stained with DAPI for 10 min. The neurons were washed three times with PBS, and then, the cell morphology was detected on an IncuCyte S3 live-cell analysis system [12,27].

### 3.7. Statistical Analysis

Only cells with neurites longer than one cell body diameter were counted. The length of the longest neurite (50–100 cells) were measured by Image J software and expressed as percentage of growth or length, relative to control (* *p* < 0.05, ** *p* < 0.01, or *** *p* < 0.001). The percentage of growth rate was calculated as follows: growth rate (%) = (length of tested group–length of control group)/control group × 100%. All data were expressed as mean ± SD. A statistical analysis was performed using IBM SPSS Statistics 20. Statistical significances were evaluated with a two-sided unpaired Student’s t-test for two-group comparisons, and a one-way ANOVA followed by the Dunnett post hoc test for multigroup comparisons. *p* < 0.05 was considered statistically significant [12,28].

## 4. Conclusions

Fucosylated chondroitin sulfate (FCS) from the sea cucumber *Acaudina molpadioides* has been obtained (FCS*_Am_*), and its precise structure was preliminary investigated in our previous work. An unusual disaccharide branch [GalNAc-(α1,2)-Fuc_3S4S_] was first found in natural FCS, and sulfated fucose Fuc_2S4S_ and Fuc_4S_ were also elucidated as the branches linked to O-3 of GlcA in the central core. In view of the novelty and variety of the branch type of FCS*_Am_*, further investigation of structure and activity were conducted in this study. From the glycosidic bond-selectively depolymerized product (dFCS*_Am_*), several size-homogenous fractions (F2–F5) with the molecular weight increasing from 1.5 kDa to 4.4 kDa were obtained by GPC separation. Furthermore, seven oligosaccharides were purified after repeat charge-separation from fraction F3. A detailed analyses of the NMR and MS spectroscopic data clearly presented their structures as hexa-, hepta-, octa-, and nonasaccharide. Combined with the oligomers including tri-, tetra-, penta-, and hexasaccharide obtained from fractions F1 and F2, the precise structure of native FCS*_Am_* was confirmed. Its CS-E-like backbone was composed of the repeating disaccharide unit [3)-GalNAc_4S6S_-(β1,4)-GlcA-(β1,], where all the GlcA residues were branched by sulfated fucose [Fuc_2S4S_ (60%) and Fuc_2S4S_ (25%)] and heterodisaccharide [GalNAc-(α1,2)-Fuc_3S4S_ (15%)]. The disaccharide branches dispersively distributed in the saccharide chain rather than clustered.

Based on the fractions and oligosaccharides with rich structural features, the effects on the cortical neuron outgrowth were evaluated. The natural FCS*_Am_* and its depolymerized products dFCS*_Am_* showed neurite outgrowth promotion and cell aggregation simultaneously. For the size-homogonous fraction (F2–F5), F2 could stimulate the outgrowth, and the promoting effects increased with the increase of molecular weight. A comparison of analyses among the purified oligosaccharides also showed that the promoting activity was positively correlated with chain length, while an optimum chain length is required for maximum growth without aggregation. In addition, the branch Fuc_2S4S_ significantly contributed to the promotion, whereas the novel disaccharide branch [GalNAc-(α1,2)-Fuc_3S4S_] did not show obvious difference in promotion or inhibition effects. The results expanded the range of FCS activity analysis and provided some guidance for drug research in neuro-systemic diseases.

## Figures and Tables

**Table 1:** ^1^H (800 MHz) and ^13^C (200 MHz) NMR data for **9**, **10**, **11, 12** and **13a** in D_2_O.

Comp.	Residue	*δ*_H_, ppm							*δ*_C_, ppm							
		H1	H2	H3	H4	H5	H6	H8	C1	C2	C3	C4	C5	C6	C7	C8
**9**	A’	5.09	4.27	3.89	4.00	4.17	3.78/3.74	2.09	102.4	52.1	71.0	71.5	74.6	64.3	178.0	25.1
	II	5.40	**4.04**	*4.74*	*4.91*	4.34	1.31		101.1	**75.1**	*76.7*	*81.9*	69.2	18.6		
	dU	4.90	3.84	**4.53**	5.74				105.8	73.3	**78.9**	109.4	149.6	171.6		
	A	4.62	4.12	**4.18**	*4.98*	4.06	*4.30/4.25*	2.06	102.4	54.2	**78.7**	*79.2*	74.5	*70.2*	177.8	25.3
	I	5.68	*4.50*	4.15	*4.86*	4.96	1.38		99.6	*77.9*	69.3	*83.9*	69.1	18.5		
	U	4.60	3.73	**3.79**	**3.94**	3.79			105.8	77.1	**80.3**	78.1	79.0	178.0		
	rA	3.70	4.27	**4.28**	*4.50*	4.41	*4.15*	2.01	62.5	54.4	**77.8**	*80.6*	70.3	*72.4*	177.0	24.8
**10**	dI	5.51	*4.44*	4.15	*4.70*	4.36	1.31		99.0	*77.7*	69.2	*83.5*	69.1	18.4		
	dU	4.91	3.90	**4.51**	5.76				105.9	73.1	**79.1**	109.1	149.8	171.6		
	dA	4.61	4.12	**4.15**	*4.98*	4.04	*4.31/4.20*	2.06	102.6	54.1	**78.7**	*79.1*	75.0	*70.1*	177.7	25.3
	I	5.70	*4.49*	4.16	*4.88*	4.94	1.39		99.3	*77.8*	69.3	*83.9*	69.1	18.6		
	U	4.48	3.67	**3.76**	**3.96**	3.70			106.6	76.6	**79.9**	**78.1**	79.7	177.7		
	rA	4.78	4.07	**4.01**	*4.83*	4.06	*4.30/4.20*	2.06	103.5	54.3	**78.9**	*79.1*	74.6	*70.5*	177.7	25.3
	rI	5.22	*4.46*	4.21	*4.74*	4.59	1.33		101.3	*78.0*	69.3	*83.8*	69.5	18.7		
	rU	3.83	4.05	**4.05**	**4.17**	4.32			65.1	73.3	**80.6**	**83.0**	75.0	180.0		
**11**	dI	5.50	*4.42*	4.14	*4.69*	4.35	1.30		99.0	*77.7*	69.1	*83.4*	69.0	18.4		
	dU	4.90	3.88	**4.50**	5.75				105.9	73.1	**79.1**	109.1	149.8	171.6		
	dA	4.60	4.11	**4.16**	*4.96*	4.04	*4.31/4.18*	2.05	102.5	54.1	**78.7**	*79.1*	74.6	*70.5*	177.7	25.3
	III	5.40	3.80	4.04	*4.77*	4.88	1.37		101.3	71.1	71.5	*83.9*	69.0	18.7		
	U	4.48	3.62	**3.69**	**4.00**	3.69			106.3	76.6	**80.2**	**78.3**	79.8	177.7		
	rA	4.79	4.04	**4.04**	*4.81*	4.04	*4.28/4.18*	2.05	103.4	54.3	**78.2**	*79.1*	74.7	*70.5*	177.7	25.3
	rI	5.22	*4.44*	4.20	*4.73*	4.58	1.32		101.2	*78.0*	69.2	*83.8*	69.5	18.7		
	rU	3.80	4.03	**4.03**	**4.15**	4.32			65.1	73.4	**80.7**	**82.8**	74.9	180.1		
**12**	dIII	5.20	3.80	4.01	*4.62*	4.33	1.29		101.4	71.1	71.2	*83.4*	69.1	18.6		
	dU	4.94	3.88	**4.41**	5.75				105.5	72.9	**78.8**	109.5	149.3	171.7		
	dA	4.61	4.11	**4.16**	*4.95*	4.06	*4.30/4.20*	2.06	102.5	54.2	**78.7**	*79.0*	75.0	*70.0*	177.4	25.3
	I	5.69	*4.49*	4.16	*4.86*	4.93	1.37		99.3	*77.8*	69.1	*83.9*	69.0	18.6		
	U	4.47	3.66	**3.75**	**3.95**	3.69			106.6	76.5	**80.0**	**78.2**	79.7	177.7		
	rA	4.77	4.02	**4.02**	*4.82*	4.03	*4.29/4.18*	2.06	103.5	54.3	**78.9**	*79.0*	74.6	*70.5*	177.7	25.3
	rI	5.22	*4.45*	4.21	*4.74*	4.58	1.32		101.3	*78.0*	69.3	*83.8*	69.5	18.7		
	rU	3.79	4.03	**4.04**	**4.17**	4.31			65.1	73.3	**80.7**	**83.0**	74.9	180.0		
**13a**	dI	5.51	*4.43*	4.10	*4.69*	4.35	1.30		98.9	*77.7*	69.1	*83.5*	69.1	18.4		
	dU	4.90	3.89	**4.52**	5.76				106.0	73.1	**79.0**	109.0	149.9	171.6		
	dA	4.61	4.17	**4.16**	*4.97*	4.06	*4.38/4.30*	2.06	102.6	54.2	**78.7**	*79.0*	74.5	*70.5*	177.8	25.4
	A’	5.28	4.25	3.88	4.11	4.18	3.78/3.74	2.09	100.5	52.1	70.9	71.4	74.5	64.3	177.8	25.4
	II	5.45	**4.15**	*4.69*	*5.15*	4.98	1.43		101.2	**74.7**	*78.1*	*82.2*	69.2	18.7		
	U	4.50	3.62	**3.80**	**3.96**	3.71			105.6	76.3	**80.4**	**78.0**	80.1	177.8		
	rA	4.80	4.05	**4.13**	*4.76*	4.06	*4.29/4.20*	2.05	103.3	54.3	**76.2**	*79.1*	74.8	*70.5*	178.1	25.3
	rI	5.22	*4.46*	4.21	*4.75*	4.64	1.33		101.0	*78.0*	69.2	*83.8*	69.5	18.7		
	rU	3.80	4.02	**4.05**	**4.19**	4.33			65.1	73.5	**80.5**	**82.8**	75.1	180.1		

Values in *italic* and **bold** type indicate positions of sulfation and glycosylation, respectively.

**Table 2:** Negative-ion ESI-MS of oligosaccharides **8**–**13**.

Comp.	Molecular Ions	*m/z*		Molecular Formula	Mw
		Observed	Calculated		
**8**	[M−3Na]^3−^ [M−4Na + H]^3−^	614.2876 606.9607	614.2869 606.9596	C_40_H_54_O_54_N_2_S_8_Na_10_	1913.2097
**9**	[M−3Na + H]^2−^ [M−4Na + H]^3−^[M−3Na]^3−^	1023.4730 674.6529 681.9800	1023.4737 674.6527 681.9800	C_48_H_67_O_59_N_3_S_8_Na_10_	2116.4037
**10**	[M−4Na + H]^3−^ [M−5Na + H]^4−^ [M−6Na + H]^5−^	789.6083 586.4597 464.5697	789.6094 586.4597 464.5700	C_52_H_69_O_70_N_2_S_10_Na_13_	2461.5350
**11**	[M−3Na]^3−^ [M−4Na]^4−^	762.9568 566.4712	762.9571 566.4705	C_52_H_70_O_67_N_2_S_9_Na_12_	2359.4962
**12**	[M−4Na + H]^3−^ [M−5Na + H]^4−^ [M−6Na + H]^5−^	755.6310 560.9767 444.1845	755.6298 560.9751 444.1822	C_52_H_70_O_67_N_2_S_9_Na_12_	2359.4962
**13a**	[M−4Na + H]^3−^	857.3014	857.3025	C_60_H_82_O_75_N_3_S_10_Na_13_	2664.7290
	[M−5Na + H]^4−^ [M−4Na]^4−^	637.2300 642.7250	637.2296 642.7251		
**13b**	[M−3Na]^3−^	830.6498	830.6503	C_60_H_83_O_72_N_3_S_9_Na_12_	2562.6902
	[M−4Na + H]^3−^ [M−4Na]^4−^	823.3222 617.2408	823.3229 617.2404		

**Table 3:** Statistical analysis of neurite outgrowth assays performed on FCS*_Am_*, dFCS*_Am_*, and size-homogeneous fractions (%, mean ± SD).

Treatment	Mw (kDa)	Conc. (μg/mL)	Neurite Length (% of Control)
F2	~1.5 *^a^*	50	152.10 ± 21.18
F3	~2.5^*a*^	50	179.70 ± 6.83 **
F4	~3.5 *^a^*	50	188.60 ± 9.83 **
F5	~4.4^*a*^	50	218.50 ± 13.24 **
dFCS*_Am_*	4.13 *^b^*	50	150.40 ± 13.07
FCS*_Am_*	51.7^*b*^	50	148.90 ± 9.13 *
CS-E	72^*c*^	50	112.00 ± 27.06
Control	/	-	100 ± 3.10

* *p* < 0.05, ** *p* < 0.01 vs. control. *^a^* Data was calculated according to the number of structural unit. *^b^* Data was from our previous work [23]. *^c^* Data was from the Certification of Analysis provided by the manufacturer.

**Table 4:** Statistical analysis of neurite outgrowth assays performed on the oligosaccharides (μm, mean ± SD).

**Conc. (μM)**	**1**	**3**	**4**	**5**	**6**	**8**
4.4	116.30 ± 18.76	130.80 ± 12.35	138.80 ± 21.85	129.40 ± 14.98	144.70 ± 13.40	146.10 ± 5.16
13.3	140.20 ± 13.45	146.00 ± 16.81	141.20 ± 16.85	147.10 ± 15.19	175.60 ± 9.89 ***	177.90 ± 16.96 ***
40	149.10 ± 16.68	159.80 ± 4.43 **	164.30 ± 10.43 **	171.80 ± 23.78 ***	186.40 ± 4.10 ***	189.70 ± 18.06 ***
**Conc. (μM)**	**9**	**10**	**11**	**12**	**13**	
4.4	146.00 ± 12.51	167.80 ± 25.99	133.60 ± 14.12	160.30 ± 20.22 **	160.80 ± 7.96 **	
13.3	206.00 ± 6.56 ***	202.90 ± 10.72 ***	157.10 ± 11.42 *	187.60 ± 12.16 ***	196.20 ± 15.59 ***	
40	193.40 ± 18.15 ***	206.60 ± 25.99 ***	203.20 ± 11.42 ***	217.40 ± 22.29 ***	216.60 ± 31.12 ***	

* *p* < 0.05, ** *p* < 0.01, or *** *p* < 0.001 vs. control.

## Data Availability

All data presented in this study are available from the corresponding author on reasonable request.

## Supplementary Materials

The following are available online at https://www.mdpi.com/article/10.3390/md20100653/s1, Figure S1. 1H/13C NMR spectra of oligosaccharide **9**; Figures S2–S4. HSQC spectrum and signal assign-ments of oligosaccharide **10**–**12**; Figure S5. MS spectra and signal assignments of oligosaccharides **8**–**13**.
