# Supplementary material for: Branched Chondroitin Sulfate Oligosaccharides Derived from the Sea Cucumber Acaudina molpadioides Stimulate Neurite Outgrowth
Source: Mar Drugs. 2022 Oct 21;20(10):653. doi: 10.3390/md20100653 (PMC9605008; doi:10.3390/md20100653)
Supplement: Supplementary file 1 [file marinedrugs-20-00653-s001.zip › marinedrugs-1976270-supplementary-update.pdf]

Supporting Information

Branched Chondroitin Sulfate Oligosaccharides Derived from the Sea Cucumber *Acaudina molpadioides* Stimulate Neurite Outgrowth

Weili Wang [1,2,†], Hui Mao [1,2,†], Sujuan Li [1,2], Longlong Zhang [3], Lian Yang [1], Ronghua Yin [1,4,*] and Jinhua Zhao [1,4,*]

[1] State Key Laboratory of Phytochemistry and Plant Resources in West China, Kunming Institute of Botany, Chinese Academy of Sciences, Kunming 650201, China;
[2] University of Chinese Academy of Sciences, Beijing 100049, China
[3] State Key Laboratory for Conservation and Utilization of Bio-Resources, Key Laboratory for Microbial Resources of the Ministry of Education, School of life Sciences, Yunnan University, Kunming 650091, China;
[4] School of Pharmaceutical Sciences, South-Central Minzu University, Wuhan 430074, China
* Correspondence: yinrh77@163.com (R.Y.); zhaojhscu@163.com (J.Z.)
† These authors contributed equally to this work.

Table of contents

Figure S1. $^{1}H/^{13}C$ NMR spectra of oligosaccharide **9**.

Figures S2-4. HSQC spectrum and signal assignments of oligosaccharide **10–12**.

Figure S5. MS spectra and signal assignments of oligosaccharides **8–13**.

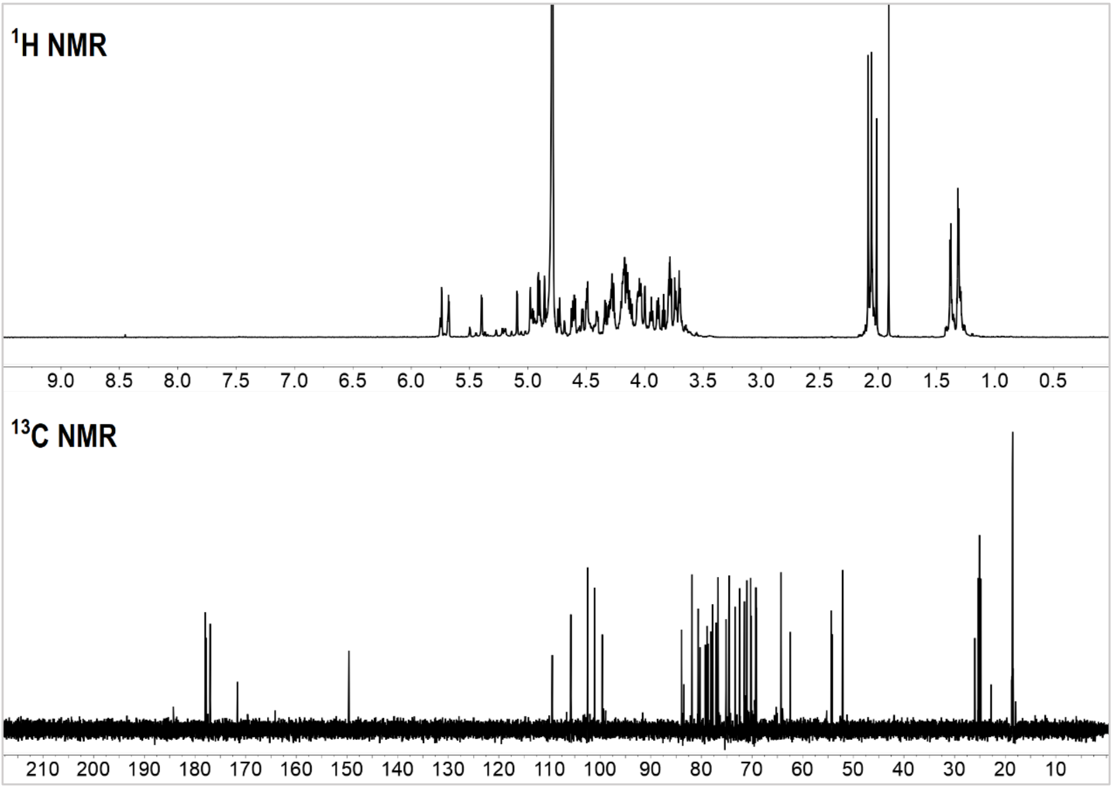

Figure S1. ^{1}H/^{13}C NMR spectra of oligosaccharide **9**.

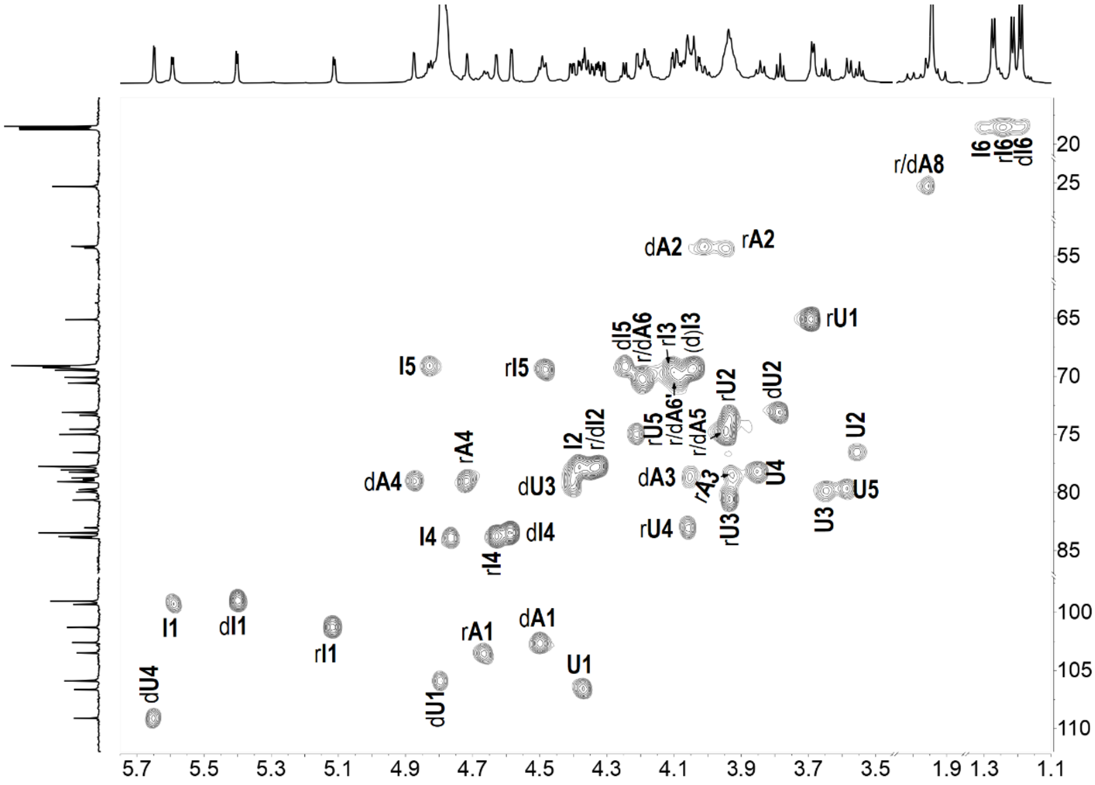

Figure S2. HSQC spectrum and signal assignments of oligosaccharide **10**.

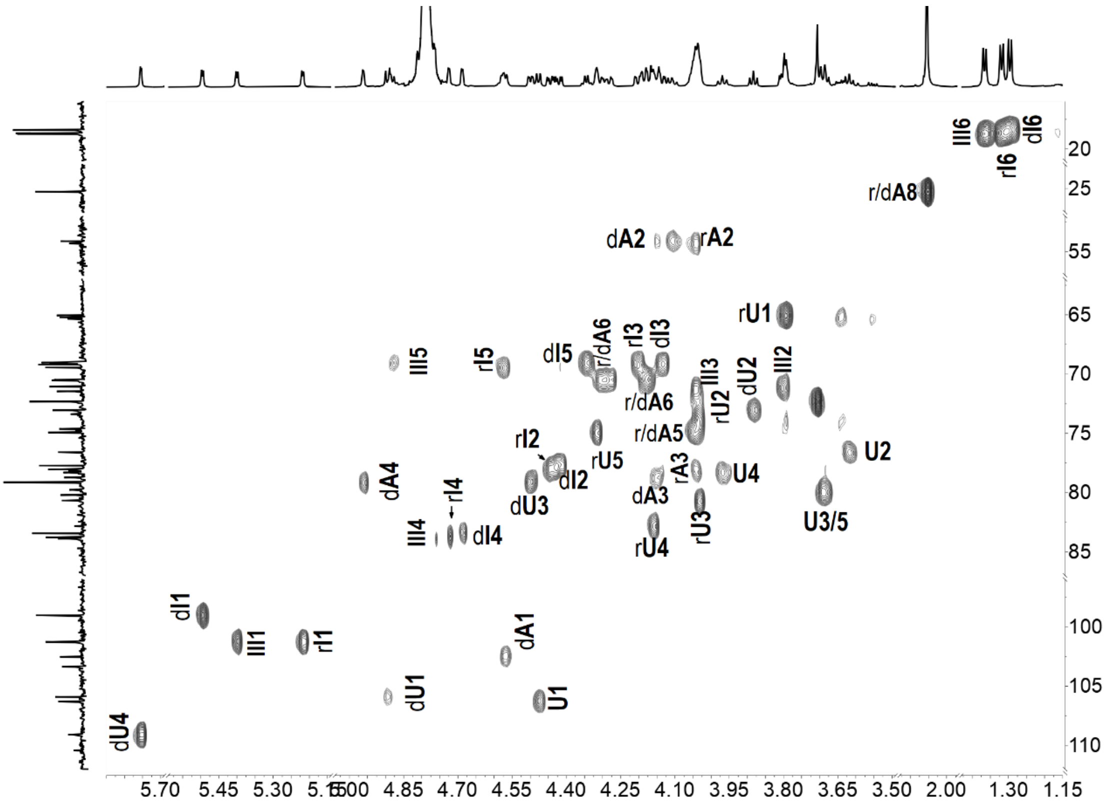

Figure S3. HSQC spectrum and signal assignments of oligosaccharide **11**.

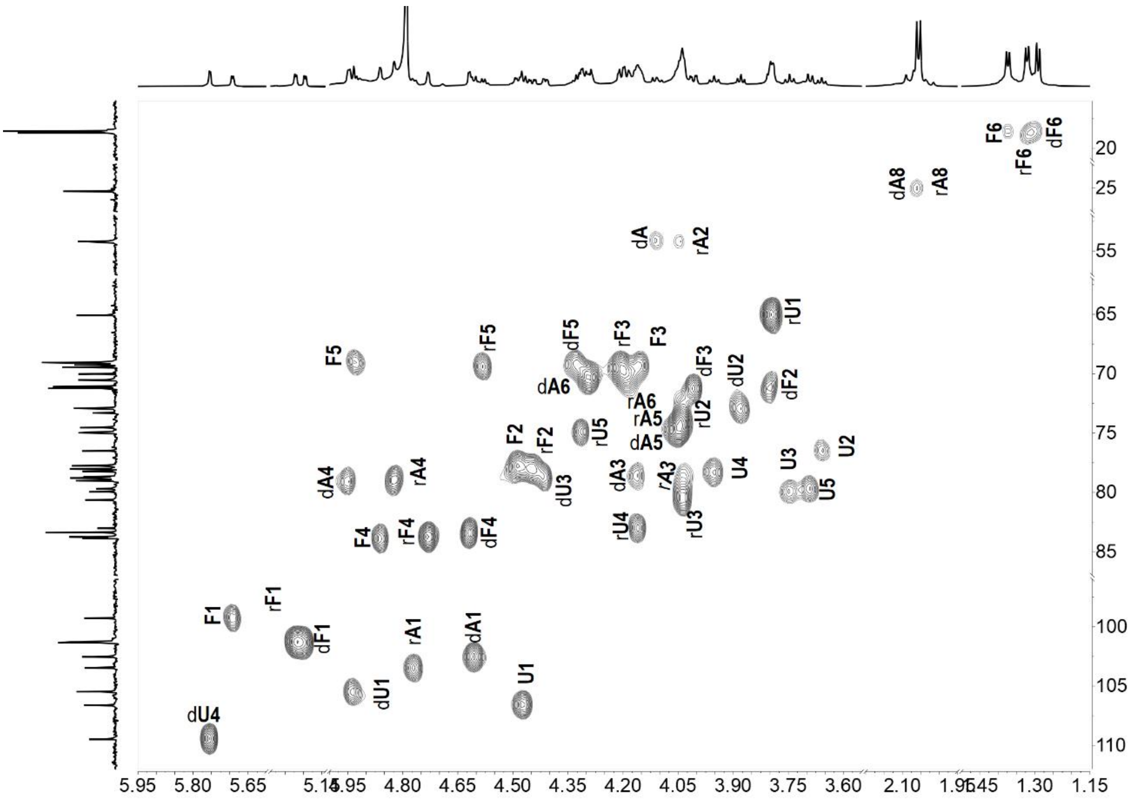

Figure S4. HSQC spectrum and signal assignments of oligosaccharide **12**.

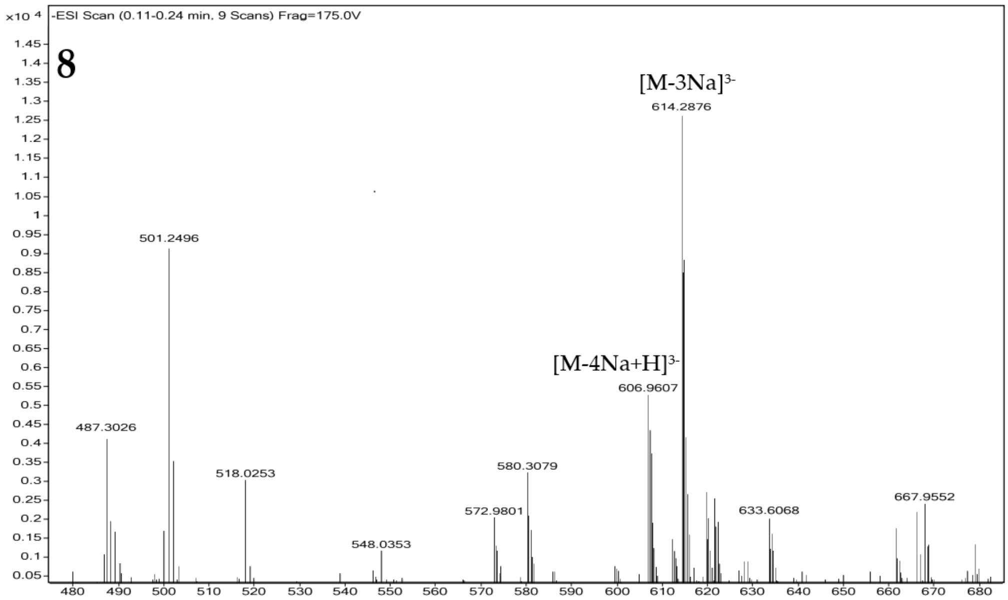
x10 4
-ESI Scan (0.11-0.24 min. 9 Scans) Frag=175.0V
8
[M-3Na]3-
614.2876
501.2496
[M-4Na+H]3-
606.9607
487.3026
518.0253
580.3079
572.9801
548.0353
633.6068
667.9552

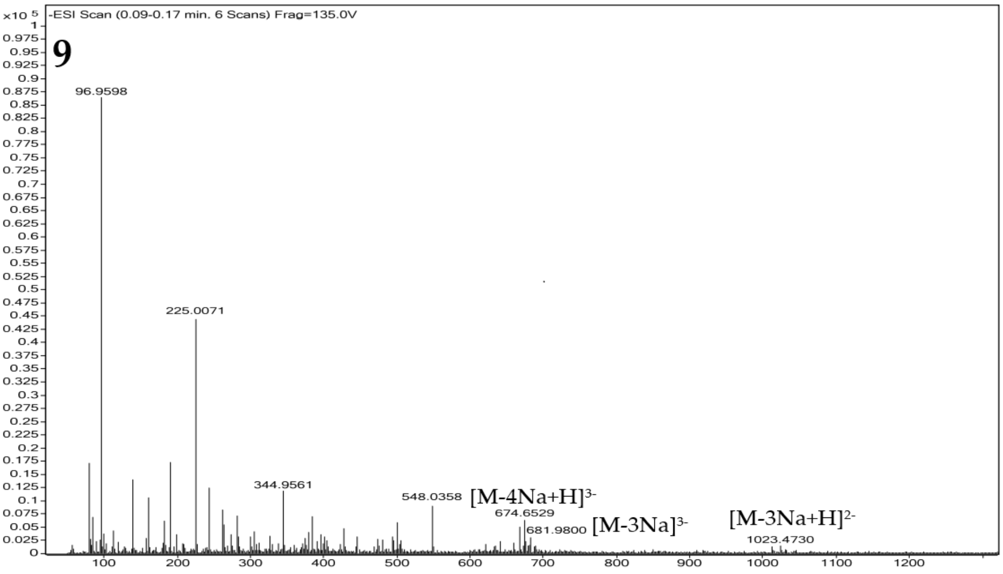
x10 5
-ESI Scan (0.09-0.17 min. 6 Scans) Frag=135.0V
9
96.9598
225.0071
344.9561
548.0358
[M-4Na+H]3-
674.6529
681.9800
[M-3Na]3-
[M-3Na+H]2-
1023.4730

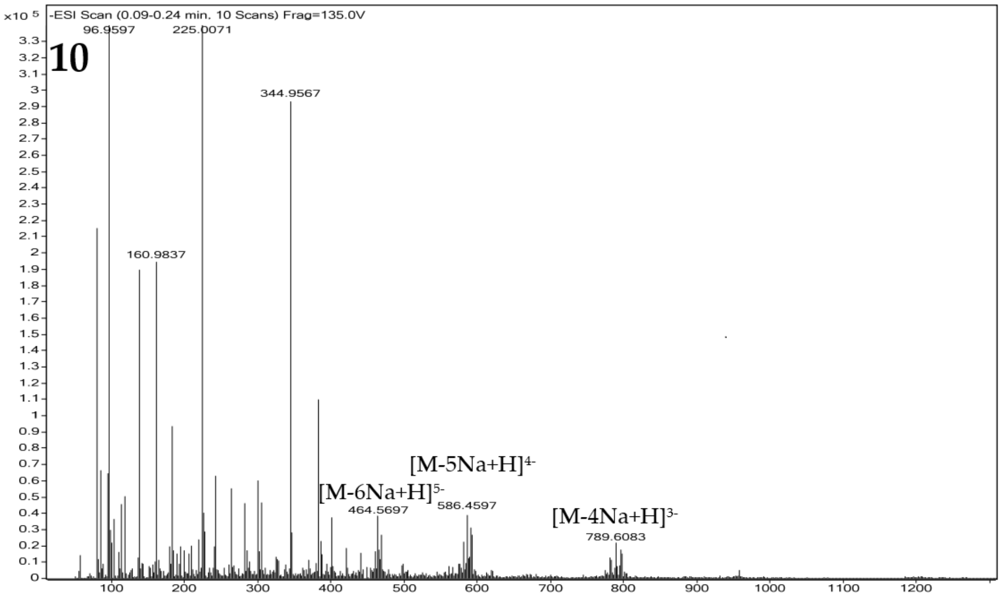
x10 5
-ESI Scan (0.09-0.24 min. 10 Scans) Frag=135.0V
10
96.9597
225.0071
344.9567
160.9837
[M-5Na+H]4-
[M-6Na+H]5-
464.5697
586.4597
[M-4Na+H]3-
789.6083

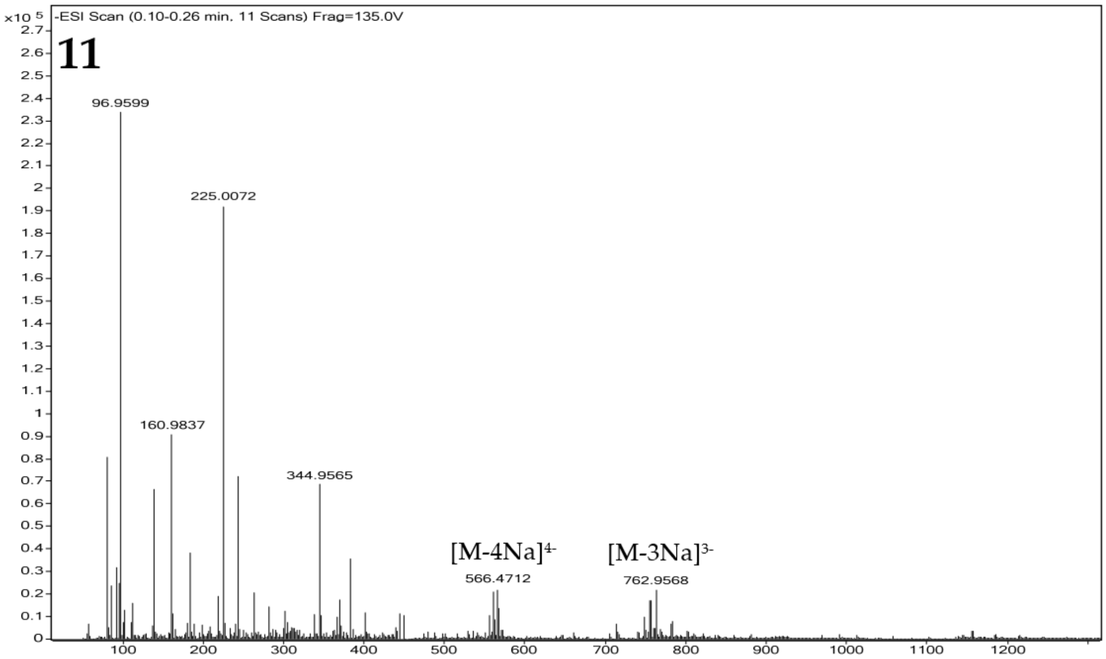

-ESI Scan (0.10-0.26 min, 11 Scans) Frag=135.0V
x10 5
11
96.9599
225.0072
160.9837
344.9565
[M-4Na]4-
566.4712
[M-3Na]3-
762.9568

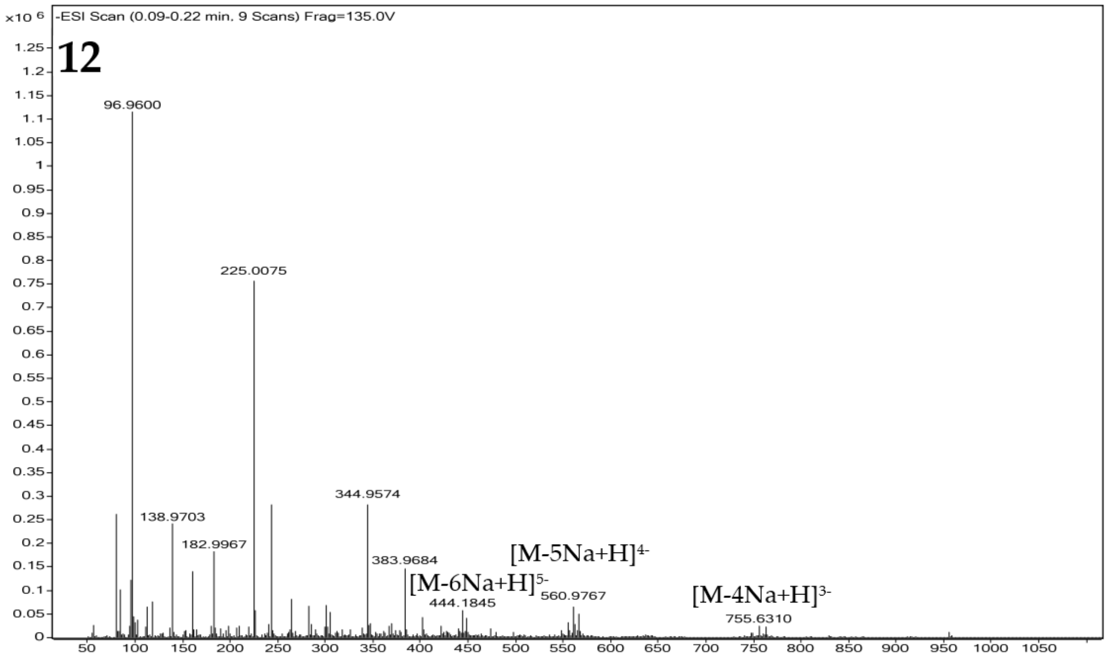

-ESI Scan (0.09-0.22 min, 9 Scans) Frag=135.0V
x10 6
12
96.9600
225.0075
344.9574
138.9703
182.9967
383.9684
[M-5Na+H]4-
[M-6Na+H]5-
444.1845
560.9767
[M-4Na+H]3-
755.6310

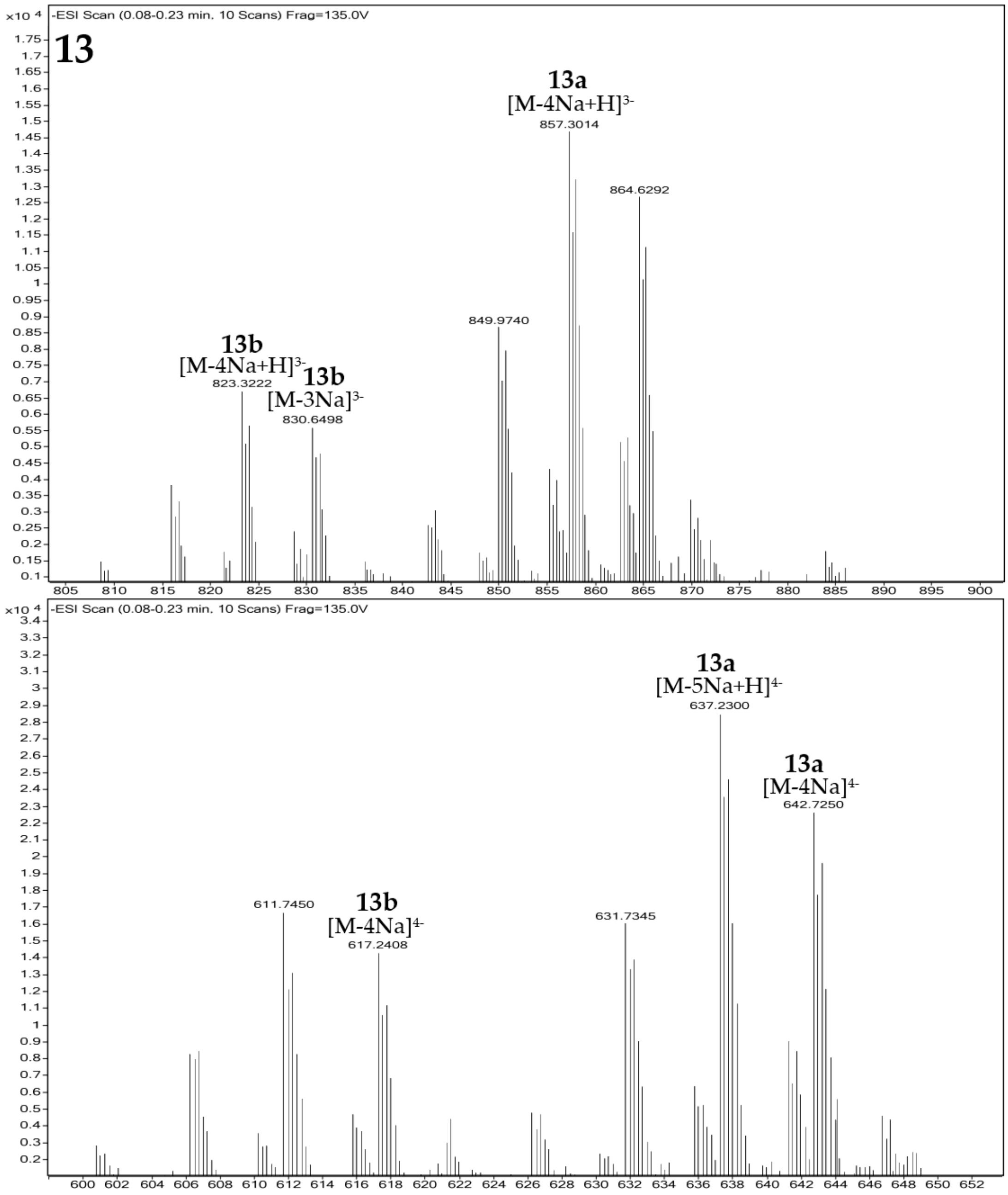

Figure S5. MS spectra and signal assignments of oligosaccharides **8–13**.
